# Obstructive Sleep Apnea and Inflammation: Proof of Concept Based on Two Illustrative Cytokines

**DOI:** 10.3390/ijms20030459

**Published:** 2019-01-22

**Authors:** Leila Kheirandish-Gozal, David Gozal

**Affiliations:** Child Health Research Institute, Department of Child Health, University of Missouri School of Medicine, Columbia, MO 65201, USA

**Keywords:** sleep apnea, inflammation, cytokines, excessive daytime sleepiness, sleep

## Abstract

Obstructive sleep apnea syndrome (OSAS) is a markedly prevalent condition across the lifespan, particularly in overweight and obese individuals, which has been associated with an independent risk for neurocognitive, behavioral, and mood problems as well as cardiovascular and metabolic morbidities, ultimately fostering increases in overall mortality rates. In adult patients, excessive daytime sleepiness (EDS) is the most frequent symptom leading to clinical referral for evaluation and treatment, but classic EDS features are less likely to be reported in children, particularly among those with normal body-mass index. The cumulative evidence collected over the last two decades supports a conceptual framework, whereby sleep-disordered breathing in general and more particularly OSAS should be viewed as low-grade chronic inflammatory diseases. Accordingly, it is assumed that a proportion of the morbid phenotypic signature in OSAS is causally explained by underlying inflammatory processes inducing end-organ dysfunction. Here, the published links between OSAS and systemic inflammation will be critically reviewed, with special focus on the pro-inflammatory cytokines tumor necrosis factor α (TNF-α) and interleukin 6 (IL-6), since these constitute classical prototypes of the large spectrum of inflammatory molecules that have been explored in OSAS patients.

## 1. Obstructive Sleep Apnea Syndrome (OSAS) and Morbidity

Obstructive sleep apnea syndrome (OSAS) is characterized by recurring events of partial or complete upper airway collapse during sleep, resulting in altered alveolar ventilation, intermittent hypoxemia along with increased respiratory efforts and intra-thoracic negative pressure swings that frequently lead to arousal and therefore perturb sleep continuity and result in fragmented sleep architecture. Obesity is a major risk factor of OSAS [1,2,3,4,5,6,7], and both of these conditions impose adverse neurocognitive, mood, behavioral, cardiovascular, and metabolic consequences in both children and adults. In addition, as the awareness and consequently the frequency of diagnosing OSAS have increased, a large list of additional OSAS-associated morbidities has been reported, including chronic kidney disease, erectile dysfunction, ocular conditions, Alzheimer disease, nocturia, and even cancer in adults, while in children enuresis and bruxism are frequent adverse consequences [8,9,10,11,12,13,14,15,16,17,18,19,20,21,22,23,24,25,26,27,28,29,30,31]. Efforts are ongoing to develop new and more effective therapies for OSAS based on underlying mechanisms promoting upper airway collapsibility during sleep [32,33]. However, the current first line treatment for OSAS in children is surgical adenotonsillectomy (T&A) and in adults consists of the administration of nasal continuous positive airway pressure (CPAP) therapy, both of which can result in transformative outcomes [34]. 

Although OSAS is associated with a 2–3-fold increased risk of developing a large spectrum of end-organ morbidities, not all patients with OSAS manifest evidence of any given end-organ dysfunction. The variability of the clinical phenotype has prompted intense investigation, especially focused around the role of systemic inflammation in OSAS-associated morbidities, particularly those affecting neurocognitive, cardiovascular, or metabolic functions [35,36,37,38,39,40,41,42]. However, exploration of systemic inflammatory pathways as candidate biomarkers failed to identify distinctive panels of circulating inflammatory markers that accurately differentiated between at-risk OSAS pediatric patients from those who appear to be less susceptible [20,43,44,45,46,47,48,49,50,51,52]. These are problematic findings since many of the OSAS morbidities are usually silent, progressive, and potentially reversible during earlier stages but can slowly progress to become either irreversible or only partially reversible over time [53,54]. Moreover, because the interactions between OSAS and obesity are multifaceted, it is difficult to identify exclusive OSAS biomarkers, since obesity can usually alter the expression and circulating levels of such biomarkers, and vice versa [55,56,57,58,59,60,61,62]. In addition, treatment of OSAS has been associated with worsening obesity [63], which can potentially dampen the response of inflammatory biomarkers to treatment. To this effect, we will not attempt to disentangle the effects of obesity and OSAS on inflammation in light of the intricate and reciprocal interactions between these two chronic low-grade inflammatory conditions. However, we should also emphasize that the link between OSAS and systemic inflammation is robust and has been the focus of a very large and diverse number of studies over now two decades aimed at elucidating the causal relationships between OSA and inflammatory pathways, as well as identifying potential biomarkers that point to either the presence of OSAS or of its associated morbidities [64,65,66,67,68,69,70,71]. 

## 2. Tumor Necrosis Factor-α

Tumor necrosis factor-α (TNF-α) is a classic pro-inflammatory cytokine that has been implicated in the regulation of sleep [72,73,74,75]. Systemic administration of TNF-α promotes the probability and depth of physiological sleep states, particularly enhancing the time spent in non-rapid eye movement (NREM) sleep phase. In addition, TNF-α levels exhibit circadian patterns, are enhanced following sleep deprivation, and the targeted disruption of TNF-α receptors or their inhibition in the CNS will result in the suppression of spontaneous NREM sleep [62]. Of note, TNF-α will traditionally lead to the activation of NF-κB pathways that in turn activate nitric oxide synthase, cyclooxygenase 2, and adenosine A1 receptors, all of which are implicated in sleep regulation [72,73,74,75]. Sleep fragmentation paradigms mimicking the sleep disruption that characterizes OSAS induces substantial up-regulation of TNF-α expression in the CNS and other tissues in mice, along with increased sleep propensity along with cognitive and mood disturbances, similar to those occurring in OSAS, even in the absence of restricted sleep duration [76,77]. Moreover, treatment with a TNF-α neutralizing antibody in wild-type mice subjected to fragmented sleep, or when the same sleep perturbation is applied to double TNF-α receptor null mice, results in marked attenuation of the increased sleep propensity as well as in attenuation of the cognitive and behavioral disturbances induced by sleep disruption [78,79]. In addition to the intrinsic causal link between sleep perturbations and TNF-α demonstrated in both murine and human experiments, similar studies in mice focused on the chronic intermittent hypoxia that characterizes OSAS further demonstrated the recruitment of TLR-4-NF-κB pathways along with increased cellular and extracellular levels of TNF-α, thereby lending further credence to the pathophysiological role of this cytokine in the context of OSAS [78,79,80,81,82,83,84,85,86,87,88,89,90,91,92].

In addition to OSAS or its intrinsic components fostering a pro-inflammatory state and manifesting as increased circulating levels of TNF-α, it is also possible that the reciprocal relationships might favor the emergence of upper airway dysfunction or of other mechanisms that facilitate the onset of OSAS. For example, intermittent hypoxia can generate inflammatory processes in the carotid body, which then translate into altered immunoregulation as well as perturbations in control of breathing that may facilitate the propensity for respiratory instability during sleep [93,94,95,96,97,98,99]. Furthermore, although specific studies are lacking in relation to upper airway musculature, increases in TNF-α in the context of other conditions (e.g., obesity) may promote muscle dysfunction and therefore enhance the likelihood of upper airway dysfunction [100,101,102].

**Figure 1:**
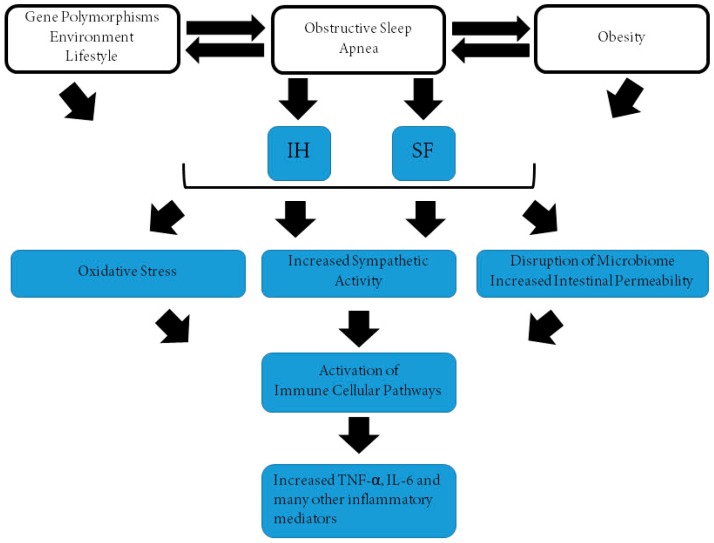
Schematic diagram illustrating the putative interactions between obstructive sleep apnea, obesity, and both genetic, environmental, and lifestyle factors, ultimately leading to a cascade of pathophysiological pathways that result in increased systemic inflammation as illustrated by increased levels of TNF-α and IL-6.

Since excessive daytime sleepiness (EDS) is a common clinical feature of OSAS in adults [79] and in obese children [80,81,82], an association between EDS and TNF-α has been proposed in the context of OSAS [103,104]. The cumulative evidence from such studies (Table 1) indicates that circulating TNF-α levels are inconsistently elevated in either adult patients with OSA independent of obesity or in children. Indeed, out of the 37 studies published to date in adults, 27 showed higher levels of TNF-α, with eight not detecting evidence of increased TNF-α, circulating concentrations in OSAS, and an additional two studies reporting equivocal findings (Table 1). In this context, some of the differences may be due to an insufficient number of subjects being recruited, disparities in the severity of OSAS, discrepant distribution of concurrent obesity, as well as potential ethnic differences. Notwithstanding, only few of the studies evaluated TNF-α levels before and after treatment, an important consideration when evaluating associations between diseases and potential biomarkers. Such problems were all the more apparent in the pediatric studies, whereby only 3 of 10 studies showed elevated TNF-α concentrations in OSAS, with five studies showing negative findings and two studies being equivocal in their conclusions. However, despite such inconsistent findings, a recent meta-analysis indicated that plasma TNF-α levels are overall increased in OSAS, albeit modestly, and are being even proposed as a biological marker of EDS in OSAS [105]. Indeed, Nadeem and colleagues reported that standardized pooled mean differences (SPMD) were 1.77 for high sensitivity C-reactive protein (hs-CRP), 1.03 for TNF-α, 2.16 for IL-6, 4.22 for IL-8, 2.93 for ICAM, 1.45 for selectins, and 2.08 for VCAM [105]. In another report that included pre- and post-CPAP treatment in adults, the SPMDs (95% confidence interval [CI]) for hs-CRP, IL-6, IL-8, and TNF-α were 0.452 (95% CI, 0.252–0.651), 0.299 (95% CI, 0.001–0.596), 0.645 (95% CI, 0.362–0.929), and 0.478 (95% CI, 0.219–0.736) in pre- and post-CPAP therapy, respectively, further reinforcing the assumption that OSAS is a pro-inflammatory state that is responsive to adherent CPAP treatment [106]. Of note, the presence of a single nucleotide polymorphism-308 in the TNF-α gene differs among OSAS patients versus controls and appears to be clustered among those patients with concurrent EDS [107,108,109,110,111,112]. In a pilot study, treatment with etanercept to reduce TNF-α activity was accompanied by significant reductions in EDS in adult patients with OSAS [113]. Taken together, there is substantial variability across OSAS patients regarding the presence of elevated systemic concentrations of TNF-α, suggesting that the direct effect of OSAS may be strongly modulated by factors that either enhance (e.g., TNF-α gene polymorphisms) or attenuate (e.g., diet, physical activity) such associations, thereby supporting the initial conceptual framework that the magnitude of systemic inflammation in the context of OSAS operates as a major determinant of the morbid consequences of this disease (Figure 1). Future studies specifically examining these relationships, and utilizing a composite panel of inflammatory biomarkers rather than isolated cytokine levels may facilitate improved delineation of personalized risk assessments. 

## 3. Interleukin 6

Interleukin 6 (IL-6) belongs to the so-called IL-6 family of cytokines. All of its members, which include cardiotrophin-1, oncostatin M, leukemia inhibitory factor, cardiotrophin-like cytokine, ciliary neurotrophic factor, and the interleukins 11, 27, 30, and 31, bind to the glycoprotein 130 (gp130) as a β-receptor to activate intracellular signaling cascades. These cascades generally consist of homo- or heterodimers of gp130 in combination with other cytokine receptors [161]. Plasma levels of the inflammatory biomarker hs-CRP, whose expression is IL-6 dependent in liver, predict the risk of vascular disease in addition to other disease conditions such as diabetes and cognitive function deterioration. In the context of OSAS, hs-CRP levels tend to be elevated in afflicted children, independent of the degree of obesity [38]. Adipose tissue inflammation is induced by intermittent hypoxia and by chronic sleep fragmentation, can result in elevated IL-6 release [162,163,164,165,166,167,168], and may cross-talk with endothelial cells via adipocyte-derived mediators such as IL-6 to promote NF-κB-dependent endothelial dysfunction [169]. 

Furthermore, IL-6 plasma levels correlate with endothelial dysfunction, arterial stiffness, and the magnitude of subclinical atherosclerosis and are also predictive of incident type 2 diabetes and obesity [170,171]. The marked overlap between the repertoire of conditions in which IL-6 is either a risk biomarker or an actual effector of morbidity and the OSAS morbid consequences suggest that IL-6 may serve as a reliable reporter of either the presence of OSAS or of the risk of OSAS-associated morbidities. This assumption is further buttressed by the fact that intermittent hypoxia, one of the hallmark characteristics of OSAS, induces polarization of macrophages along with increased production of IL-6 [172]. Biopsies of adipose tissue and blood samples in obese patients with and without OSAS, revealed substantial increases in tissue expression and circulating levels of a variety of pro-inflammatory cytokines, including IL-6, and such changes were markedly attenuated by six months of CPAP therapy [159]. Interestingly, adult patients with OSAS and objective EDS documented by reduced sleep latencies exhibited significantly elevated daytime and nighttime IL-6 plasma levels, that were absent when no EDS was present [154]. Pooling of eight published reports in adults with OSAS revealed that plasma levels of IL-6 ranged from 1.2 to 131.66 pg/mL before CPAP treatment and significantly decreased to between 0.45 to 66.04 pg/mL after CPAP treatment (*p* < 0.05), but they also indicated that there was significant inter-individual heterogeneity [155]. Similar heterogeneity was detected in IL-6 levels in children with OSAS [156,173,174,175,176] and may be related to genetic variance for both IL-6 and CRP genes [176]. In a recent meta-analysis of gene polymorphisms for IL-6 and corresponding plasma levels, the authors concluded that IL-6 gene polymorphism -174 G/C, but not -572 G/C, is associated with OSAS risk in adults and that IL-6 levels are globally increased in OSAS but that CPAP treatment does not consistently reduce elevated IL-6 levels [176], the latter being possibly related to underlying obesity or concurrent cardiovascular or metabolic disease [143,176]. In children with OSAS, IL-6 plasma levels were also generally higher and were significantly reduced after T&A surgery [156]. 

## 4. Conclusions

The examination of two classic prototypic inflammatory cytokines such as IL-6 and TNF-α provides confirmation that OSAS in both adults and children promotes a persistently low intensity inflammatory state. However, substantial heterogeneity is present in the detectable manifestation of OSAS-associated inflammatory processes, indicating substantial modulation by genetic factors as well as by environmental and lifestyle influences. Notwithstanding, the cumulative findings are congruent with the assumption that increases in inflammatory markers in OSAS patients likely reflect the presence of underlying silent or overt end-organ morbidity. Future studies aimed at unraveling reliable and specific inflammatory biomarker panels that can confidently discriminate who are the patients at higher risk for OSAS-induced morbidities should not only allow for an improved understanding of the pathophysiology of OSAS morbidities but also facilitate the implementation of precision medicine interventions among OSAS patients [135,136]. Such studies should obviously and pre-emptively avoid the pitfalls of many of the published studies and therefore be sufficiently powered, adopt a pre- post-treatment intervention model, and carefully consider many of the potential confounders that are likely to be operationally associated in the context of a chronic disease such as OSAS.

## Figures and Tables

**Table 1:** Summary of published studies examining TNF-α serum concentrations in adults and children with obstructive sleep apnea syndrome (OSAS) over the last 15 years. Green rows indicate significant findings, orange rows indicate negative findings, and yellow rows reflect equivocal results.

Reference (First Author, Year)	Number of Subjects	Association With	TNF-α Levels Associated With	Effect of Treatment (Tx)	Comments	TNF-α Levels Are Increased in OSA Yes/No/Equivocal
**ADULTS**						
[114]	50 OSA and 50 controls	OSAS severity Insulin resistance		No Tx		YES
[115]	120 OSA; 40 controls	Carotid atherosclerosis	OSA severity cIMT	CPAP reduced TNF-α levels		YES
[116]	30 OSA 10 controls			MAD reduced TNF-α levels		YES
[117]	100 OSA 50 controls	Atherosclerosis cIMT; pulse wave velocity	No	CPAP for 3 months reduced TNF-α levels		YES
[118]	25 OSA undergoing uvulopalatal flap (UPF) surgery		No	UPF surgery reduced TNF-α levels		YES
[119]	Meta-analysis of 2857 OSA and 2115 controls	OSA severity	Yes; in mild, mild-to-moderate, moderate, moderate-to-severe, and severe OSAS, circulating TNF-alpha was higher than controls by 0.99, 1.48. 7.79, 10.08, and 8.85 pg/mL, with significant heterogeneity (I2: 91.2%, 74.5%, 97.6%, 99.0% and 98.1%)	No Tx		YES
[120]	1042 subjects from community	OSA severity Metabolic syndrome	Positive association in women and negative in men	No Tx		YES
[121]	20 obese OSA 6 non-obese OSA			Reduced cytokines after 6 months CPAP or surgery		YES
[122]	52 subjects (10 controls, 42 obese OSA)	Insulin resistance	Higher TNF-α	No Tx	Inverse relationship between IL-10, but not TNF-α and insulin resistance	YES
[66]	31 OSA and erectile dysfunction (ED) 15 OSA and no ED		Higher TNF-α plasma levels when ED present			YES
[123]	80 OSA 40 controls	cIMT	Higher TNF-α plasma levels associated with higher cIMT	No Tx		YES
[124]	22 OSA	Association with apnea-hypopnea index	Higher TNF-α plasma levels	CPAP for 3 months reduced TNF-α plasma levels		YES
[125]	363 men	ED	Higher TNF-α plasma levels when ED and OSA present	No Tx		YES
[126]	32 OSA and metabolic syndrome	Endothelial function		CPAP for 3 months reduced TNF-α plasma levels		YES
[127]	230 habitually snoring women and 170 controls	AHI ODI3%	Significant association between TNF-α levels and ODI3%			YES
[128]	66 OSA			CPAP 8 months reduced TNF-α plasma levels in men but not in women		YES
[129]	51 OSA	EDS		Upper airway surgery with 4-week follow-up showed significant reductions in TNF-α plasma levels and EDS		YES
[130]	OSA (n = 113) Hypertensive without OSA (n = 73) Hypertensive with OSA (n = 134) Controls (n = 97)		OSA patients have higher TNF-α levels	No Tx arm		YES
[131]	84 mild OSA 40 controls		OSA patients have higher TNF-α levels	No Tx arm		YES
		Monocyte production of TNF-α levels	Circulating monocytes in OSA patients have higher TNF-α levels	No Tx arm		YES
[132]	33 OSA 13 controls					
[133]	24 OSA 12 non-obese and 15 obese controls			Surgery decreased monocyte TNF-α production		
[134]	24 OSA 27 controls			CPAP for 1 month decreased monocyte production of TNF-α		
[135]	52 OSA			CPAP for 6 months (n = 32 with good adherence and 20 non-adherent). Good adherence reduced TNF-α plasma levels		YES
[136]	32 severe OSA and metabolic syndrome			CPAP adherence for 8 weeks (n = 16) reduced TNF-α plasma levels but no changes if non-adherent (<4 h/night)		YES
[137]	106 OSA 32 controls		OSA patients, particularly if concurrent obesity, have higher TNF-α levels	No Tx arm		YES
[138]	50 OSA 20 controls	EDS	OSA have higher TNF-α levels unrelated to EDS	No Tx arm		YES
[139]	27 OSA 11 controls		Higher TNF-α levels in OSA	No Tx arm		YES
[140]	43 OSA 22 controls	BMI	OSA have higher TNF-α levels unrelated to BMI	No Tx arm		YES
[141]	18 OSA 20 controls		OSA have higher TNF-α levels	No Tx arm		YES
[142]	159 OSA and no-OSA, obese and non-obese	Serum d-lactate Intestinal permeability	No significant associations	No Tx arm		NO
[143]	220 non-sleepy OSA	Coronary artery disease	No	Randomization to CPAP or no CPAP for 1 year had no effect on TNF-α levels		NO
[144]	28 OSA on CPAP 29 OSA undergoing upper airway surgery		No	No effects of either Tx on TNF-α levels		NO
[145]	391 OSA		No differences before and after treatment	CPAP for 6 months		NO
[146]	52 OSA and no-OSA obese	Metabolic syndrome	No differences in OSA with or without metabolic syndrome	No Tx arm		NO
[147]	35 OSA			CPAP for 3 months – no changes in TNF-α plasma levels		NO
[148]	43 OSA	Serum and induced sputum	Sputum TNF-α levels, but not serum levels, correlated with OSA severity	No Tx		NO
[149]	110 OSA 45 controls		No differences in TNF-α levels	No Tx arm		NO
[69]	89 OSA; 28 snorers; 26 controls	Pharyngeal lavage and plasma	Higher cytokines including TNF-α in pharyngeal lavage but not in plasma	1-year follow up CPAP—improvements in TNF-α in pharyngeal lavage		Equivocal
[150]	70 severe OSA	Hypertension	Higher TNF-α plasma levels associated with hypertension	No Tx arm		Equivocal
**CHILDREN**						
[151]	19 children	Cardiac magnetic resonance imaging (aortic blood flow velocity and left and right ventricular systolic function)	-	No Tx arm	Intra-cellular TNF-α in CD8+T cells	YES
[152]	35 children OSA	None	None	T&A reduced TNF-α at 6 months follow up		YES
[153]	298 snoring children	EDS	TNF-α significantly higher with more severe OSA and when EDS present	T&A and 3 months follow-up showed significant reductions in TNF-α		YES
[154]	164 overweight and obese children (111 controls, 28 mild OSA, 25 moderate-to-severe OSA)	OSA severity	None	No Tx arm		NO
[155]	90 controls 65 OSA	Pulse transit time (PTT)	Shorter PTT	No Tx arm		NO
[156]	392 adolescents with no OSA, mild, moderate and severe OSA	Visceral adipose tissue	None	No Tx arm		NO
[157]	24 moderate to severe OSA 22 mild OSA 22 controls	EDS	No differences in TNF-α across 3 groups; no association with EDS	No Tx arm		NO
[158]	90 obese children with OSA			T&A and 6-month follow-up showed no changes in TNF-α or IL-6		NO
[159]	47 non-obese OSA 32 controls	Cognitive function	Association with general cognitive function	No Tx arm		Equivocal
[160]	142 snoring children		TNF-α not higher in OSA but IL-6 and IL-8 elevated	No Tx arm		Equivocal

cIMT—carotid intima media thickness; CPAP—continuous positive airway pressure; MAD—mandibular advancement device; Tx—treatment; T&A—adenotonsillectomy; EDS—excessive daytime sleepiness; ODI3%—oxygen desaturation index 3%.
